# Tunable nonlinear optical properties in nanocrystalline Si/SiO_2_ multilayers under femtosecond excitation

**DOI:** 10.1186/1556-276X-9-28

**Published:** 2014-01-14

**Authors:** Pei Zhang, Xiaowei Zhang, Jie Xu, Weiwei Mu, Jun Xu, Wei Li, Kunji Chen

**Affiliations:** 1 National Laboratory of Solid State Microstructures and School of Electronic Science and Engineering, Nanjing University, Nanjing 210093, China

**Keywords:** Optical nonlinearities, Nanocrystalline Si, Multilayers, Interface state, 42.65.-k, 42.65.Jx, 42.65.Pc

## Abstract

The nonlinear optical properties of nanocrystalline-Si/SiO_2_ (nc-Si/SiO_2_) multilayers have been investigated through Z-scan technique by using a Ti-sapphire laser with 50-fs pulse duration at 800 nm as a pump laser. It is interesting to note that with increasing the annealing temperature to make the sample change from amorphous phase to nanocrystalline state, the nonlinear absorption turns the reverse saturation absorption into saturation absorption while the nonlinear optical refraction is also changed simultaneously from self-defocusing to self-focusing. We propose that the localized states at the nc-Si/SiO_2_ interfaces play the key role in the observed switching behaviors. Our results demonstrate that the tunable optical nonlinearities can be achieved by controlling the microstructures of nc-Si, which can be used as engineering different nonlinear optical devices.

## Background

Linear and nonlinear optical properties in Si-based materials have attracted much attention in the recent years since they can be potentially applied in many kinds of optoelectronic devices by using the mature Si technology [1-5]. However, bulk crystalline Si has a weak nonlinear optical effect due to the low Kerr coefficient, which will restrict its actual applications. Recently, the enhanced nonlinear optical effect in the near-infrared spectral range has been observed in nanocrystalline Si (nc-Si) films and all-optical switch as well as optical amplifier based on nc-Si has been realized [6-8]. So far, nonlinear optical properties have been observed in nc-Si films prepared by various techniques such as chemical vapor deposition (CVD) and sputtering methods. It is found that the observed nonlinear optical behaviors are strongly dependent on the film microstructures as well as the measurement conditions [9-11]. For example, Spano et al. reported the change of nonlinear refraction indices from positive to negative with changing the film composition and measurement conditions [9]. Martínez et al. fabricated nc-Si films by three different deposition techniques: e-beam evaporation, plasma-enhanced chemical vapor deposition, and low-pressure chemical vapor deposition (LPCVD), and they found that the nc-Si films prepared by LPCVD show the saturation absorption property, while the other two samples displayed the reverse saturation absorption characteristics [10]. More recently, Ma et al. observed the tunable nonlinear absorption behaviors by changing either the incident laser intensity or the bandgap of nc-Si films [11]. Therefore, it is one of the important issues to further understand the nonlinear optical properties of nc-Si films especially under the ultrafast laser excitation.

Usually, spatially confined exciton due to quantum confinement effect is considered to play a dominant role in enhanced nonlinear optical property of nc-Si film. Prakash et al. reported the size-dependent nonlinear optical coefficient, and they attributed it to the increase of oscillator strengths because of the quantum confinement-induced localization of electron–hole pairs [6]. Meanwhile, the localized defect states are also proposed to affect the nonlinear optical properties of nc-Si films. Ito et al. found that the nonlinear refractive index did not decrease monotonously with the size of nc-Si, and they believed that both the quantized electronic states and defect states contributed to the large nonlinear refractive index [12]. In our present work, we systematically studied the nonlinear optical properties of Si/SiO_2_ multilayers during the transition process from amorphous phase to nanocrystalline Si state. We found tunable nonlinear optical behaviors, reverse saturation absorption in the amorphous-phase dominant samples, and saturation absorption in the nanocrystalline-phase dominant ones, under femtosecond laser excitation. The nonlinear refraction was also simultaneously changed. We proposed that the interface states of nc-Si play the important role in the changing of nonlinear optical behaviors.

## Methods

**Figure 1:**
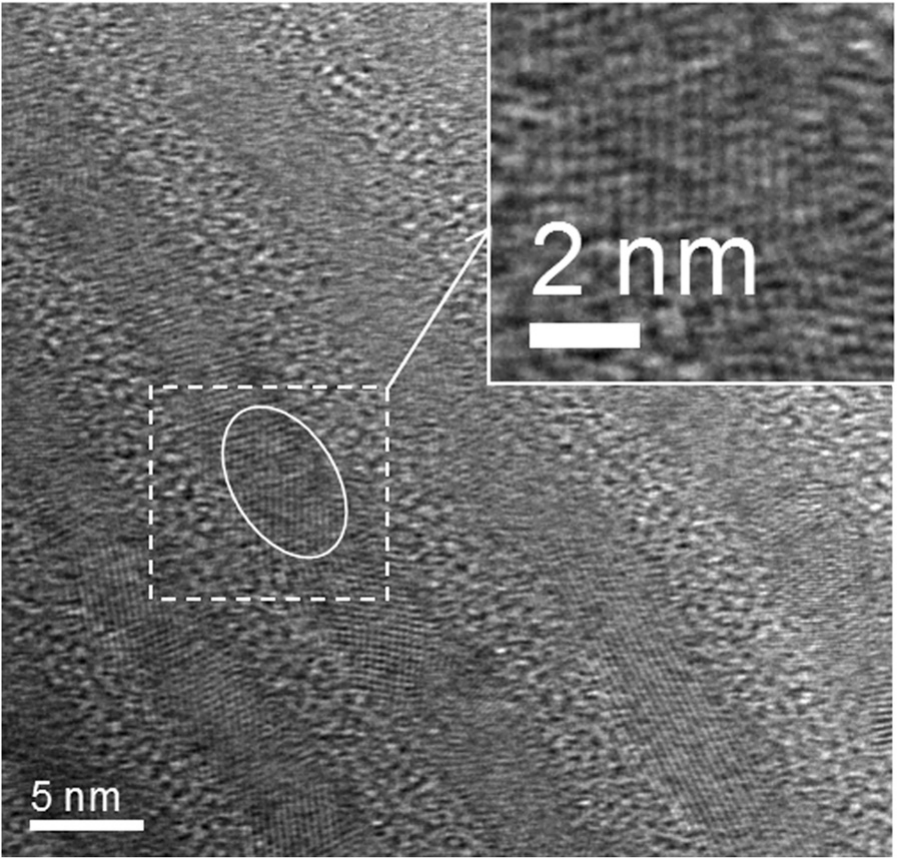
**X-TEM micrograph of sample D after annealing at 1,000°C.** The inset is the high-resolution TEM image, in which the formed nc-Si dots can be clearly identified.

The nc-Si/SiO_2_ multilayer samples with 9.5 periods studied here were obtained by thermally annealing amorphous Si/SiO_2_ stacked structure prepared in conventional plasma-enhanced chemical vapor deposition (PECVD) system. During the deposition process, the substrate temperature and radio frequency power were kept at 250°C and 50 W, respectively. The details of preparation condition can be found elsewhere [13]. As-deposited samples were dehydrogenated at 450°C for 1 h and subsequently annealed in pure N_2_ ambient to precipitate nc-Si at various temperatures (800°C to 1,000°C). Here after, we denoted the as-deposited sample, 800°C, 900°C, and 1,000°C annealed sample as samples A, B, C, and D, respectively. The microstructures of nc-Si/SiO_2_ multilayers were characterized by cross-sectional transmission electron microscopy (X-TEM) and Raman scattering spectroscopy. Figure 1 is the X-TEM image of sample D, which is clearly shown that the periodic structures are kept after annealing and nc-Si dots are formed with the size of 4 nm (as shown in the inset of Figure 1). Optical absorption spectra were measured in a spectral range of 300 to 1,000 nm using Shimadzu UV-3600 spectrophotometer (Shimadzu Corp., Kyoto, Japan), and the optical bandgap was deduced according to Tauc plots. Room-temperature photoluminescence (PL) was measured under the excitation of He-Cd laser (325 nm).

The Z-scan technique [14] was applied to measure the nonlinear optical coefficients of nc-Si/SiO_2_ multilayers. In this experiment, the excitation laser was a Ti-sapphire laser with 50-fs pulse duration at 800 nm, the repetition rate was 1 kHz. The low repetition rate is in favor of reducing the thermal accumulative effect [9]. A lens with 20-cm focal length was used to obtain Gaussian beam, the obtained beam waist was about 30 μm.

## Results and discussion

**Figure 2:**
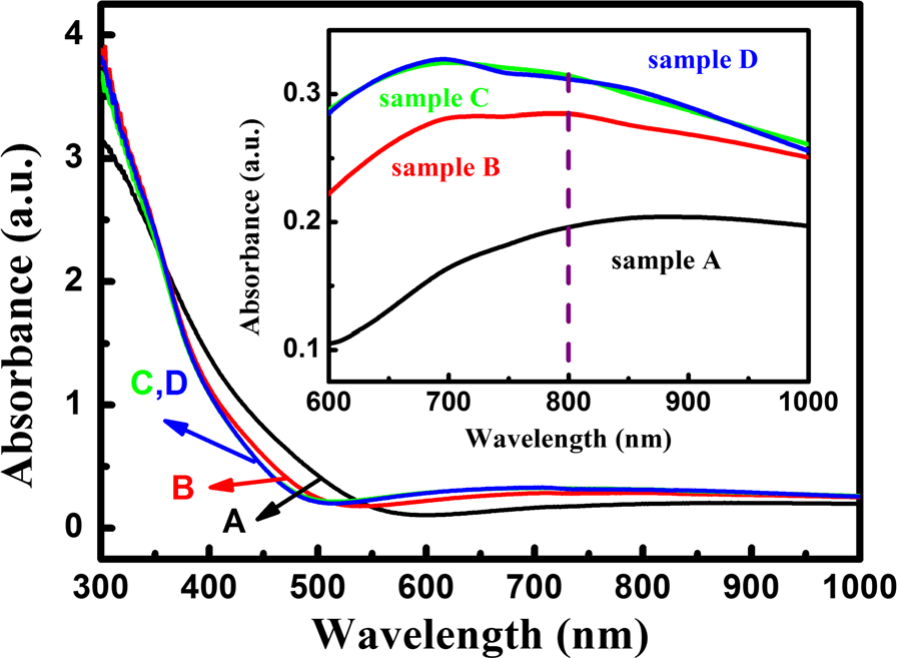
**Optical absorption spectra of samples A to D.** As-deposited Si/SiO_2_ multilayers (sample A) and samples after annealing with various temperatures (B: 800°C, C: 900°C, D: 1,000°C).

Figure 2 illustrates the absorption spectra of four samples annealing at different temperatures; it is shown that the optical absorption for the four samples is quite weak in the near-infrared range, while it becomes strong as the wavelength is shorter than 600 nm. From the absorption spectra, one can estimated the bandgap energy according to the Tauc plot. The bandgap of samples A, B, C, and D is 1.87, 2.07, 2.15, and 2.16 eV, respectively. The dash line in the inset of Figure 2 is the comparison of the absorbance at 800 nm (1.55 eV), which is lower than the optical bandgap. It is suggested that the absorption may come from the midgap states [15]. In addition, the absorption increases with increasing the annealing temperature, which means that the density of the gap states increases at higher annealing temperatures.

**Figure 3:**
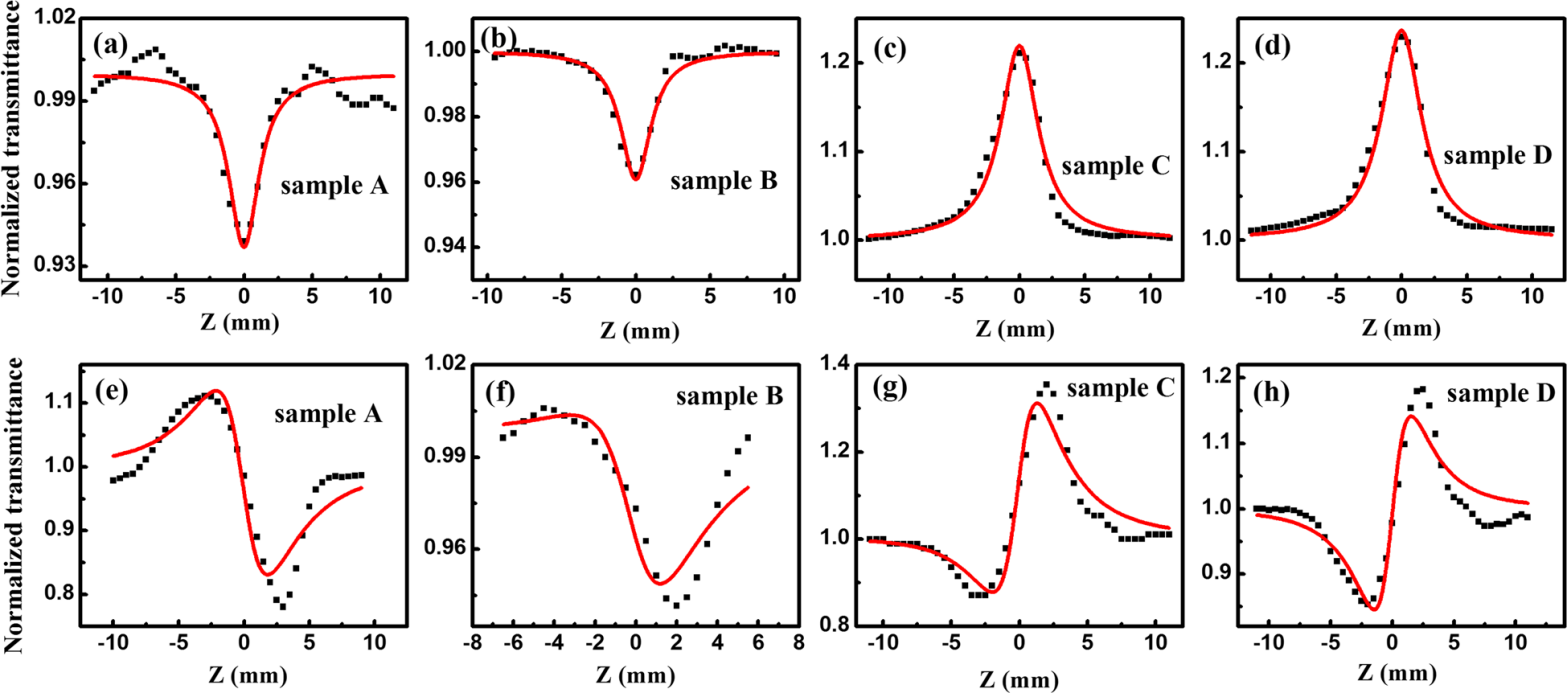
**Z-scan traces of samples A to D under laser intensity of *I***_**0**_ **= 3.54 × 10**^**11**^ **W/cm**^**2 **^**at the focal point.** The open and closed Z-scan traces are shown in **(a,b,c,d)** and **(e,f,g,h)**, respectively. Black squares are the experimental data and the solid lines are the fitting curves.

Figure 3a,b,c,d,e,f,g,h shows the normalized Z-scan transmittance traces of samples A to D under the laser intensity *I*_0_ = 3.54 × 10^11^ W/cm^2^; Figure 3a,b,c,d is measured in the open aperture configuration while Figure 3e,f,g,h is measured in the closed aperture configuration. It is interesting to find that both the nonlinear absorption (NLA) and nonlinear refraction (NLR) change obviously from sample A to sample D. The reverse saturation absorption (RSA) characteristics are observed in samples A and B, since they show the dip at the focal point as given in Figure 3a,b, while the saturation absorption (SA) can be identified in samples C and D as they show the peak at the focal point. It indicates that the NLA coefficient *β* changes from the positive value to the negative one. In the closed aperture configuration, both samples A and B exhibit peak-to-valley processes, whereas the other two samples show the valley-to-peak behaviors, which suggests that the NLR coefficient *n*_2_ changes from negative value to positive one.

Firstly, we will discuss the changes of NLA from samples A to D. Sample A is as-deposited amorphous Si/SiO_2_ multilayers which clearly shows the RSA characteristic measured by Z-scan technique in the open aperture configuration. The similar result was also reported previously in amorphous Si films, and it is originated from the two photon absorption process [9]. The nonlinear absorption coefficient *β* can be calculated from the measured transmittance data according to the following equation by considering the two-photon absorption process [14]:

(1) $$T=1-\frac{1}{2\sqrt{2}}\frac{\beta I_{0}L_{\mathrm{eff}}}{x^{2}+1},$$

where *x* = *z*/*z*_0_, $z_{0}=k\omega_{0}^{2}/2$ is the Rayleigh diffraction length, *z* is the sample position from the focal point, *I*_*0*_ is the excitation intensity at the focal point, $L_{\mathrm{eff}}=\frac{1-e^{-\alpha_{0}L}}{\alpha_{0}}$ indicates the effective thickness of the sample, *α*_0_ is the linear absorption coefficient, and *L* is the real thickness. The calculated *β* is 7.0 × 10^-8^ cm/W, which is comparable to the value reported previously [12].

**Figure 4:**
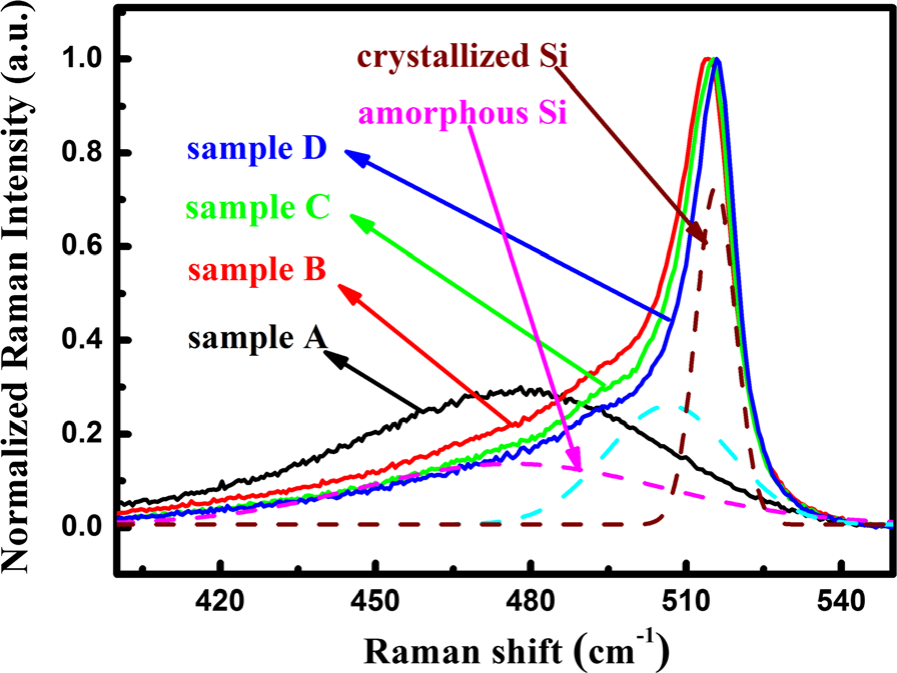
**Normalized Raman spectra of samples A to D.** As-deposited Si/SiO_2_ multilayers (sample A) and samples after annealing with various temperatures (B: 800°C, C: 900°C, D: 1,000°C). The Raman spectra of sample D are decomposed by three components: the crystallized phase component peaked at 516 cm^-1^ (wine dash line), transition phase 506 cm^-1^ (cyan dash line), and the amorphous component peaked at 480 cm^-1^ (magenta dash line).

For sample B after 800°C annealing, it is noted that the α-Si sublayers begin to be crystallized as revealed by Raman spectra, as shown in Figure 4, and the crystallinity is about 61%. The NLA coefficient is reduced to 4.2 × 10^-8^ cm/W, which can be explained in terms as two factors. First, we find that the optical bandgap slightly increases from 1.89 eV (sample A) to 2.07 eV (sample B), which means that the density of states at the same energy level in conduction band decreases due to the enlargement of the bandgap; therefore, the number of absorbed photon via two photon absorption (TPA) process is reduced at the same incident intensity. Second, due to the formation of nc-Si dots after annealing, part of incident photons can be absorbed to excite carriers from the valence band to localized states existing in the interfacial region of nc-Si and SiO_2_ layers, which may reduce the two photon absorption process between valence and conduction band. Consequently, the nonlinear absorption *β* is reduced in sample B.

**Figure 5:**
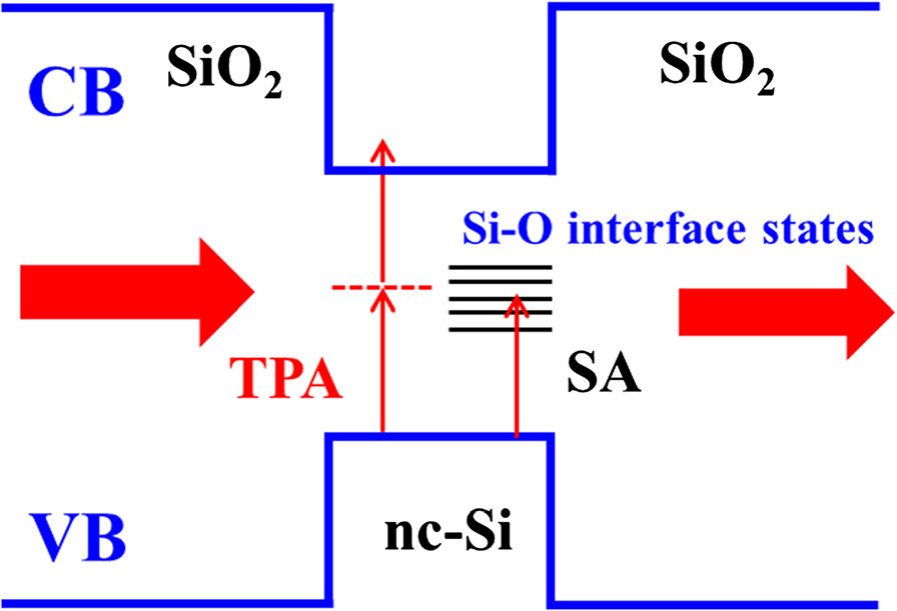
**The schematic diagram of nonlinear optical response processes.** The nonlinear optical response includes two-photon absorption (TPA) and phonon-assisted one-photon absorption via interface states for our samples.

It is interesting to find that the nonlinear absorption coefficient becomes negative in samples C and D due to the SA process. As shown in Figure 4, it is found that with increasing the annealing temperature, the relative Raman signal of crystallized Si phases (centered at 516 and 506 cm^-1^) becomes stronger compared to that of amorphous Si phase (approximately 480 cm^-1^) and the band width becomes narrower; meanwhile, the Raman peak of nc-Si shifts toward the higher wave number, which indicates that samples C and D are further crystallized after annealing at higher temperature due to the formation of more nc-Si. The high density of nc-Si dots results in much more interface states of nc-Si dots, which in consistent with the linear absorption properties, as shown in Figure 2. Therefore, the single photon transition from valence band to the interface states has been a main route to generate nonlinear absorption behaviors and the two photon absorption process can be neglected in this case. Consequently, the SA occurs to cause the negative nonlinear absorption coefficient. The observed phenomenon is similar with the optical nonlinearity of Au nanoparticles [16], for which both the two photon absorption and saturation absorption can be observed. By taking into account the SA process, the nonlinear absorption coefficient *β* can be expressed by Equation 2 [17]:

(2) $$\beta =-\frac{\alpha_{0}}{I_{S}+I_{0}},$$

where *β* is the saturation absorption coefficient and *I*_s_ is the saturation irradiance. The *β* for samples C and D is -2.3 × 10^-7^ and -2.5 × 10^-7^ cm/W, respectively. The SA process was previously reported in Si-based materials. Ma et al. [11] observed the SA in nc-Si/H films with the *β* in the order of -10^-6^ cm/W. They attributed the SA to the phonon-assisted one photon absorption process, in which the band-tail states acted as a crucial role in the observed NLA response. López-Suárez et al. [17] also observed the changes from RSA to SA in Si-rich nitride films with increasing the annealing temperature. The calculated *β* was -5 × 10^-8^ cm/W when nc-Si dots were formed. Since a pump laser with *λ* = 532 nm was used in their case, they suggested that the one-photon resonant absorption between the valence and conduction band resulted in the NLA characteristic. In our case, the pump wavelength is *λ* = 800 nm, which is far below the bandgap; we attribute the obtained SA to the one photon-assisted process via the localized interface states of nc-Si dots. Figure 5 is the schematic diagram of nonlinear optical response processes. Both TPA process and SA process co-exist in our samples (samples B to D). The competitions between TPA and SA determine the ultimate nonlinear optical absorption property. It is noted that the SA process is associated with the interface states in formed nc-Si. For sample B which is annealed at relatively low temperature, the two-photon absorption process induces the RSA associated with the nonlinear optical response of free carriers as in the case of sample A. When the annealing temperature increases, the more nc-Si dots are formed and the localized states existing in the interfacial region between nc-Si and SiO_2_ layers gradually dominate the nonlinear optical response. The one-photon absorption between the valence band and the localized states occurs in samples C and D, which ultimately results in the SA process.

**Figure 6:**
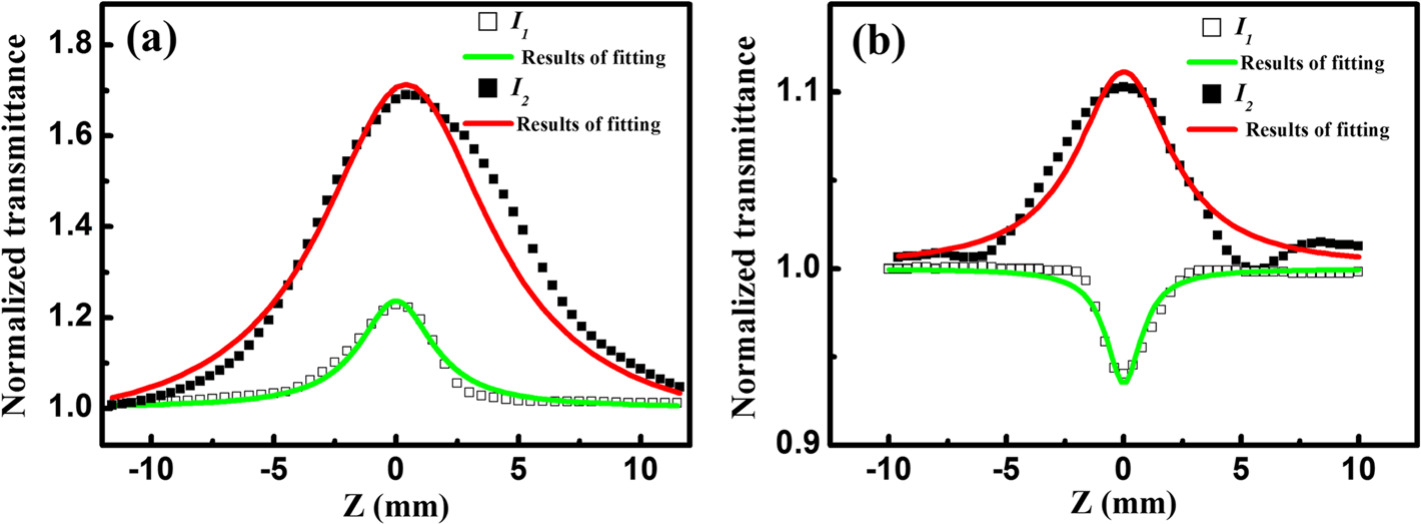
**Open aperture Z-scan traces of samples D and E. (a)** Sample D and **(b)** sample E under two laser intensity, *I*_1_ = 3.54 × 10^11^ W/cm^2^ (open square) and *I*_2_ = 3.54 × 10^12^ W/cm^2^ (full square). The solid lines are the fitting curves of the experimental data.

**Figure 7:**
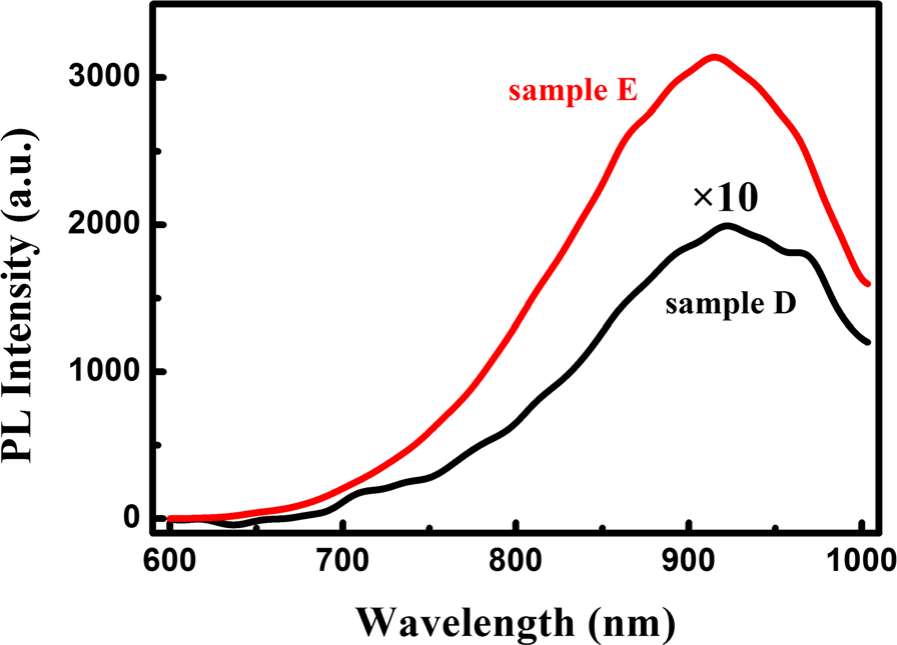
The PL spectra of sample D (black line) and sample E (red line).

In order to further understand the role of interface states in optical nonlinearity of nc-Si/SiO_2_ multilayers, we fabricate the nc-Si with small size of 2.5 nm (sample E) and investigate the NLA with the change of excitation intensity. The intensity-dependent nonlinear optical properties of amorphous Si and nc-Si-based films have been reported previously. López-Suárez et al. [17] found that the TPA process dominated the nonlinear absorption in amorphous silicon nitride films under the various excitation intensities from 2 × 10^9^ to 18 × 10^9^ W/cm^2^ though the nonlinear optical coefficient changed. Spano et al. [9] studied the variety of nonlinear absorption coefficient *β* in nc-Si films with changing the excitation intensities in a range of 1 to 5 × 10^12^ W/cm^2^; they found that TPA process dominated the nonlinear optical process under the various laser excitation intensities and the *β* decreased as increasing the excitation power. It was explained in term of the banding filling effect at high pumping power if the TPA process dominated the nonlinear optical absorption process. However, the different intensity-dependent optical nonlinearities are observed in sample E in our case. As shown in Figure 6a,b, the NLA of sample E changes from RSA to SA with increasing the excitation intensity. However, sample D keeps the SA characteristic with changing the excitation intensity while the transmittance increased, as shown in Figure 6a. As mentioned before, the SA process is sensitive to the density of interface states. For sample with small-sized nc-Si, the more interface states are introduced due to the larger surface-to-volume ratio. We also measured the PL properties of samples D and E as displayed in Figure 7 to illustrate it. It is clear to find that the sample E displays stronger PL intensity than sample D, and a broad luminescence band in the range of 700 to 1,000 nm was observed, which was attributed to the interface state-related recombination and radiative recombination in the previous work [13]. The more interface states introduced in the gap, the larger the saturation irradiance *I*_s_ will be. When the excitation intensity (*I*_1_ = 3.54 × 10^11^ W/cm^2^) is lower than the *I*_s_, the TPA dominates the NLA. Whereas, when the excitation intensity (*I*_2_ = 3.54 × 10^12^ W/cm^2^) is higher than the *I*_s_, the SA process appears and the TPA is suppressed. However, there are still two small valleys at the wings of the open aperture transmission trace, suggesting the TPA and SA processes co-exist, which is consistent with our model proposed in Figure 5.

Then, we will discuss the NLR behaviors in our samples. Accompanying with the change of NLA, the NLR characteristics are also tunable as shown in Figure 3e,f,g,h. Samples A and B show the negative nonlinear refraction index (*n*_2_) while samples C and D have the positive nonlinear refractive index. We calculated the *n*_2_ from the measured closed aperture transmittance data by using Equation 3 [18]:

(3) $$T=1+\frac{4x\Delta \Phi_{0}}{\left(x^{2}+9\right)\left(x^{2}+1\right)}-\beta I_{0}L_{\mathrm{eff}}\frac{\left(x^{2}+3\right)}{\left(x^{2}+9\right)\left(x^{2}+1\right)},$$

where *Δ*Φ_0_ = *k*_0_*n*_2_*I*_0_*L*_eff_ represents the nonlinear phase change. The nonlinear refraction index *n*_2_ of sample A is -3.34 × 10^-12^ cm^2^/W. Spano et al. also reported the negative nonlinear refraction *n*_2_ in the order of 10^-13^ cm^2^/W which is one order of magnitude lower than that of our sample [9]. The enhanced nonlinear optical refraction can be attributed to the strong free carrier nonlinearity in our multilayers sample via the two-photon absorption process as we discussed before. The nonlinear refractive index *n*_2_ in sample B is reduced to about -0.56 × 10^-12^ cm^2^/W, which is consistent with the reduced two-photon absorption process due to the enlargement of optical bandgap and the formation of nc-Si. However, for samples C and D, the positive nonlinear refractive index is obtained suggesting that different nonlinear optical process dominates the nonlinear response, the obtained *n*_2_ of samples C and D are 4.94 × 10^-12^ and 3.47 × 10^-12^ cm^2^/W, respectively. It is worth mentioning that we also measured the *n*_2_ from pure SiO_2_ layer pumped under similar condition in order to exclude the contribution of SiO_2_ layers. The calculated *n*_2_ is 1.4 × 10^-16^ cm^2^/W, which is much lower than that of Si/SiO_2_ multilayers. It is suggested that the enhanced optical nonlinearity is mainly resulted from the ultrathin Si layers. As debated before, the SA is obtained in samples C and D, and we attributed it to the existence of interface states between the nc-Si and SiO_2_ layers. Takagahara et al. theoretically predicted that excitons localized at disorders or impurities could increase its oscillator strength, which led to the large optical nonlinearity [19]. It was reported that the electrical field building up by the charges trapped at the nc-Si/SiO_2_ interface states would enhance the optical nonlinear process [20]. In our proposed model, the interface states between nc-Si and SiO_2_ layers can also localize the excitons to suppress the two photon absorption process, which can result in the enhanced nonlinear optical refraction index as obtained in our case.

## Conclusions

In summary, we observed the tunable NLA and NLR response in Si/SiO_2_ multilayers during the transition process from the amorphous to nanocrystalline phases under femtosecond excitation at 800 nm. We suggested that the two-photon absorption process dominates in the samples mainly containing amorphous Si phases, while the phonon-assisted one-photon transition process between the valence band and interface states dominates the nonlinear optical properties in nc-Si/SiO_2_ multilayers. The obtained NLA coefficient *β* is about -10^-7^ cm/W and the NLR index *n*_2_ is about 10^-12^ cm^2^/W for nc-Si/SiO_2_ multilayers which is two orders of magnitude larger than bulk Si, which indicate that nc-Si/SiO_2_ multilayers can be applied into high-sensitive photonic devices such as optical switch and Q-switch laser.

## Abbreviations

Nc-Si: Nanocrystalline Si; NLA: Nonlinear absorption; NLR: Nonlinear refraction; RSA: Reverse saturation absorption; SA: Saturation absorption; TPA: Two-photon absorption; X-TEM: Cross-sectional transmission electron microscopy.

## Competing interests

The authors declare that they have no competing interests.

## Authors' contributions

PZ and JunX conceived the idea and carried out the experiments. PZ, WM, and WL participated in the preparation of the samples. PZ, XZ, WM, and JieX took part in the experiments and the discussion of the results. PZ drafted the manuscript with the instruction of JX and KC. All authors read and approved the final manuscript.
